# Transcriptome and Gene Fusion Analysis of Synchronous Lesions Reveals lncMRPS31P5 as a Novel Transcript Involved in Colorectal Cancer

**DOI:** 10.3390/ijms21197120

**Published:** 2020-09-27

**Authors:** Anna Panza, Stefano Castellana, Giuseppe Biscaglia, Ada Piepoli, Luca Parca, Annamaria Gentile, Anna Latiano, Tommaso Mazza, Francesco Perri, Angelo Andriulli, Orazio Palmieri

**Affiliations:** 1 Division of Gastroenterology, IRCCS, Fondazione Casa Sollievo della Sofferenza, viale Cappuccini 1, 71013 San Giovanni Rotondo, Italy; giuseppe.biscaglia@gmail.com (G.B.); a.piepoli@operapadrepio.it (A.P.); a.gentile@operapadrepio.it (A.G.); a.latiano@operapadrepio.it (A.L.); f.perri@operapadrepio.it (F.P.); a.andriulli@operapadrepio.it (A.A.); o.palmieri@operapadrepio.it (O.P.); 2 Unit of Bioinformatics, Fondazione IRCCS-Casa Sollievo della Sofferenza, 71013 San Giovanni Rotondo, Italy; s.castellana@operapadrepio.it (S.C.); l.parca@css-mendel.it (L.P.); t.mazza@css-mendel.it (T.M.)

**Keywords:** sporadic CRC, synchronous lesions, fusion genes, RNA-Seq, lncRNAs, MRPS31P5

## Abstract

Fusion genes and epigenetic regulators (i.e., miRNAs and long non-coding RNAs) constitute essential pieces of the puzzle of the tumor genomic landscape, in particular in mechanisms behind the adenoma-to-carcinoma progression of colorectal cancer (CRC). In this work, we aimed to identify molecular signatures of the different steps of sporadic CRC development in eleven patients, of which synchronous samples of adenomas, tumors, and normal tissues were analyzed by RNA-Seq. At a functional level, tumors and adenomas were all characterized by increased activity of the cell cycle, cell development, cell growth, and biological proliferation functions. In contrast, organic survival and apoptosis-related functions were inhibited both in tumors and adenomas at different levels. At a molecular level, we found that three individuals shared a tumor-specific fusion named MRPS31-SUGT1, generated through an intra-chromosomal translocation on chromosome 13, whose sequence resulted in being 100% identical to the long non-coding RNA (lncRNA) MRPS31P5. Our analyses suggest that MRPS31P5 could take part to a competitive endogenous (ce)RNA network by acting as a miRNA sponge or/and as an interactor of other mRNAs, and thus it may be an important gene expression regulatory factor and could be used as a potential biomarker for the detection of early CRC events.

## 1. Introduction

Sporadic colorectal cancer (CRC) is the third most prevalent human malignancy worldwide, with approximately 1.8 million new cases and 900,000 deaths per year [1]. Although the risk of developing CRC increases with age—more than 90% of cases occur in people aged 50 or older—, recent research shows that the incidence of CRC has been increasing 1–3% annually for people younger than age 50 and decreasing in older individuals [2].

Sporadic CRC represents about two-thirds of all CRC cases [3]. In morphological terms, CRC progresses as a multistep process arising from benign adenomatous polyps (adenomas) and developing into locally invasive and metastatic cancer. Colorectal adenoma involved in tumor progression is generally transformed to become malignant, but, in about 12.7% of patients with single CRC, adenoma remnants are conserved into the carcinoma. Moreover, about one fifth of patients with single CRC is found to harbor synchronous adenomas during the diagnostic colonoscopy [4,5].

In the last two decades, by using next generation sequencing (NGS) approaches, advances in the characterization of molecular mechanisms involved in colorectal carcinogenesis have been made, and the full spectrum of driver genomic alterations is still incomplete. To date, the gold standard for the detection of adenomatous polyps and carcinomas remains the colonoscopy, while surgery of primary cancer (or the defined metastasis) and chemoradiation are the best approaches for an attempted cure [6]. The early detection of premalignant lesions is the current approach intended to decrease the risk of CRCs [7]. However, cases initially undetected and those progressed to distant metastasis have a poor prognosis [8]. Understanding genomics and post-transcriptional mechanisms behind the adenoma-to-carcinoma progression is a crucial step to identify biomarkers that might be used for the early detection of polyps at high risk of cancer development.

In this context, fusion genes and epigenetic regulators (i.e., miRNAs and long non-coding RNAs) could constitute relevant pieces of the puzzle of the tumor genomic landscape as they represent emerging classes of oncogenes with biomarker potential [9,10].

Fusion genes consist of an aberrant juxtaposition of two independent genes and may originate from structural rearrangements (inter or intra chromosomal translocations, deletions, and duplications), transcription read-through of neighboring genes or cis/trans-splicing of pre-mRNA [11]. The resulting fusion RNAs (or chimeric RNAs) and proteins are characterized by an altered regulation and structure and may play a role in the tumorigenesis. Moreover, they are associated with distinct cancer subtypes [12] where they were capable of predicting prognosis, staging, and treatment approaches for more personalized medicine [6,13]. Studies on genomic rearrangements in CRC have led to discovering VTI1A-TCF7L2 and R-spondin fusions (PTPRK-RSPO3 and EIF3E-RSPO2) as essential gene fusions [14,15,16,17]. Since then, other fusion transcripts with different prevalence rates have been documented in CRC [15,18,19,20].

Long non-coding RNAs (lncRNAs) exceed 200 nucleotides in length, have emerged as biomarkers in several cancers including CRC, and represent important regulators of gene expression [10,21,22]. Recent studies have shown that lncRNAs are involved in different stages of CRC, from adenoma to invasive cancer, and that they could function as competitive endogenous RNAs (ceRNAs) in CRC progression [23].

It remains to be clarified whether different molecular signatures are distinctive of the several steps involved in the progressive evolution from the adenoma to the carcinoma sequence development. In the present study, we evaluated this issue in CRC patients with sporadic synchronous lesions (adenomas and carcinomas) by whole transcriptome sequencing (RNA-Seq) analysis.

## 2. Results

### 2.1. Identification of Chimeric RNAs by EricScript and ChimeraScan Algorithms

Chimeric RNAs were investigated in normal, polypoid and tumor tissues of eleven individuals affected by CRC using two different fusion-search algorithms widely used in the scientific community [24]. Each of these methods makes use of different strategies for finding fusion candidates, either in the evaluation of the reads spanning the potential fusion junctions and in the way gene fusions are determined. Given the different outputs of the two software packages, we filtered out fusions whose junctions were not spanned by a sufficient number of short-reads, or when the candidate fusions were not present in known datasets. A total of 623 and 121 potential chimeric RNAs were detected through EricScript and ChimeraScan, respectively. In order to select fusion genes found exclusively in both adenomas and synchronous carcinomas, a filter was applied by selecting genes associated with cancer-related Gene Ontology terms (Supplementary Table S1) and characterized by a few isoforms. The fusions identified in the normal mucosa samples were excluded. Using these specific filtering criteria, 12 tumor-specific fusions were identified and retained in further validation analyses. The selected fusion genes are reported in Table 1, together with the estimated breakpoints, types of fusion and reliability scores.

### 2.2. Fusion Junction Validation by Reverse Transcription-PCR (RT-PCR)

**Figure 1:**
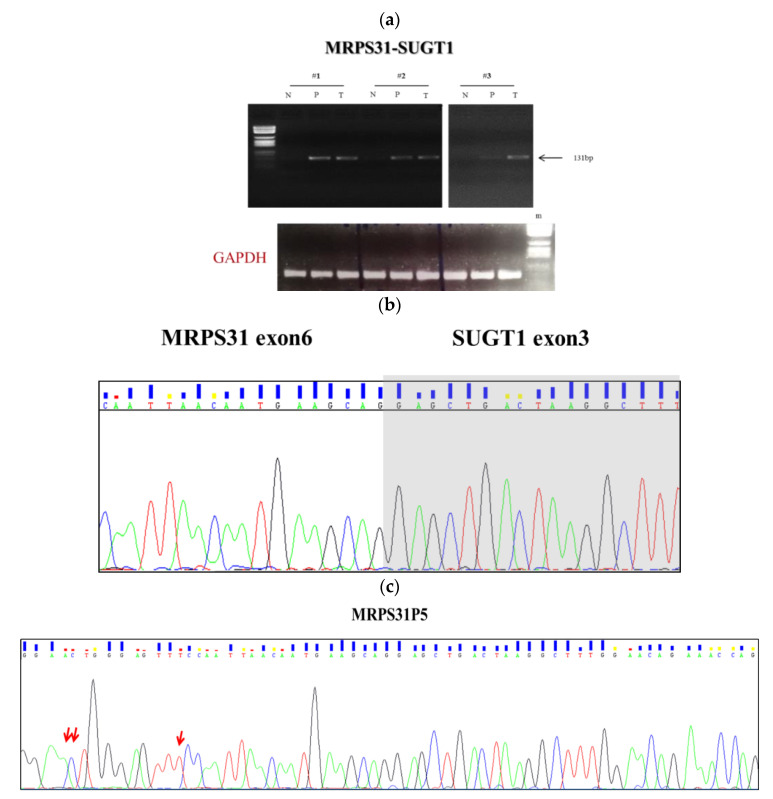
Detection of the candidate fusion gene MRPS31-SUGT1. (**a**) RT-PCR analysis of cDNA derived from pathologic tissues (P: polyp; T: tumor) and adjacent normal mucosa (N) in three CRC patients (#1, #2, #3). RT-PCR products were visualized on an agarose gel; (**b**) Sequencing analysis of the MRPS31-SUGT1 fusion transcript in patients. Sequencing electropherograms seem to reveal the fusion between exon 6 of MRPS31 and exon 3 of SUGT1 at the breakpoint; (**c**) the next check revealed that the amplicon corresponds to the MRPS31P5; the red arrows indicating the nucleotide difference between our fragment that correspond to the MRPS31P5 (100% similarity) and sequence of the exon 6 of MRPS31 (90% similarity gene.). The grouping of gels was cropped from different parts of the same gel.

For eight of 12 predicted fusion genes, namely RNF123-STAT3, PDLIM2-CCAR2, LPHN1-SUZ12, HPSE2-HSD11B2, ARSA-TNS4, EIF5AL1-MSH3, ERBB2-MIEN1, HSPE1-MOB4, we did not observe an RT-PCR product indicating a false positive call or a suboptimal RT-PCR assay. Three candidate fusions, namely PLK1-ERN2, HDAC1-MARCKSL1, and GUCY2C-PLBD1, were excluded since their RT-PCR products were also present in normal tissue samples. Of notice, the RT-PCR with junction specific primers of MRPS31 and SUGT1 genes was confirmed in both the adenomas and cancer tissues of three patients (Figure 1a). The MRPS31 gene encodes for a mitochondrial ribosomal protein implicated in membrane protein synthesis essential for oxidative phosphorylation, and it has been associated with the progression of thyroid cancer [25]. SUGT1 encodes for a highly conserved nuclear protein involved in the kinetochore function and is required for the G1/S and G2/M transitions. This protein interacts with the heat shock protein 90 (Hsp90) and is associated with the progression and abysmal prognosis in Japanese CRC patients [26].

### 2.3. Fusion Junction Validation by Sanger Sequencing

The successfully amplified fragments of the candidate MRPS31-SUGT1 were further validated through Sanger sequencing (Figure 1b,c) and BLAST alignment tool (https://blast.ncbi.nlm.nih.gov/Blast.cgi). The alignment of the PCR product showed a 90% similarity with MRPS31 (exon 6) and a 98% with SUGT1 (exon 3). As a further step, we have deeply investigated the locus predicted to be highly prone to rearrangements (Supplementary Figure S1). Among the genes with high sequence similarity located within the locus, we found a pseudogene named Mitochondrial Ribosomal Protein S31 pseudogene 5 (MRPS31P5). Further BLAST analysis between our PCR product and MRPS31P5 showed 100% similarity (data not shown).

Recently, Wu and coworkers hypothesized that MRPS31P5 gene could be a functional descendent of the HNRNPA1L2-SUGT1 chimeric RNA which is involved in the cell cycle arrest and apoptosis [27].

### 2.4. Differential Gene Expression and Functional Enrichment Analyses

Differential gene expression of tumors, adenomas and non-tumor adjacent mucosa was analyzed in the targeted RNA-Seq dataset of 1385 cancer-related genes encompassed in the Pan-Cancer Panel. This resulted in being more accurate than a typical whole RNA-Seq experiment since, by design, it improves sequence coverage and sensitivity for the detection of cancer-specific transcripts. The targeted RNA-Seq experiments produced ~10 million high-quality sequences (short-reads) for each sample.

**Figure 2:**
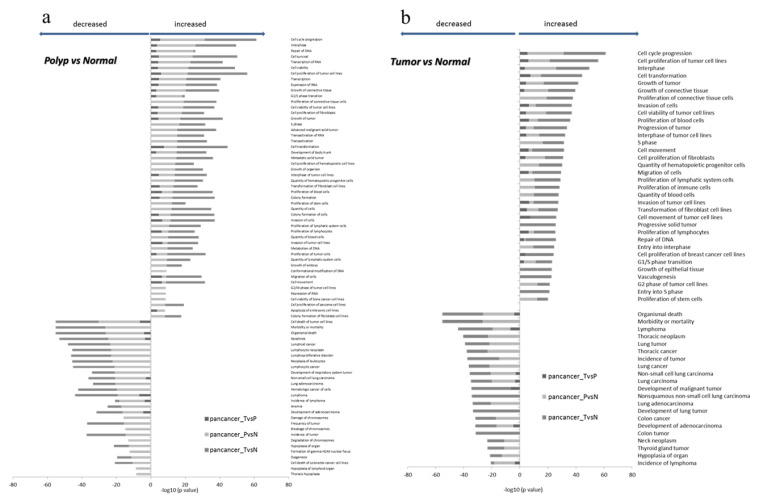
Bar chart representing the top comparing categories obtained by the functional enrichment analysis performed on differentially expressed genes. The top of decreased (Z-scores < −2) and increased functions (Z-scores > 2) was resulted by comparing polyps vs normal tissues (**a**) and tumors vs normal tissues (**b**).

Principal component analysis (PCA) showed a homogenous distribution of samples among groups, i.e., normal, polypoid and carcinoma tissues (Supplementary Figure S2). When compared with the normal mucosa, the tumor tissue was characterized by 97 down-regulated and 232 up-regulated genes, while three genes were down-regulated and 171 up-regulated in tissue samples from polyps. Comparing the results of the functional enrichment analysis performed on these genes (Figure 2), we obtained a list of 24 functions with predicted reduced functional activity (Z-scores < −2) and 43 functions with predicted increased activity (Z-scores > 2) in carcinoma samples versus normal matched tissues. We further obtained 28 decreased functions (Z-scores < −2) and 50 increased functions (Z-scores > 2) in adenoma samples versus normal tissues. Among the most activated functions, the top enriched categories included the functions related to the cell cycle, cellular development, cellular growth, and proliferation, both in tumors and in synchronous adenoma samples. As for the inhibited functions, organic survival (death, morbidity, or mortality) and the apoptosis-related functions resulted in the most enriched terms.

### 2.5. ceRNA Analysis and RNA-RNA Interactions

Concerning ceRNA analysis, we did not retrieve predicted or validated interactions between miRNAs and MRPS31P5. Consequently, we directly scanned the sequence of MRPS31P5 (NCBI Identifier: NR_051963.1) for the presence of possible miRNA recognition sites utilizing the miRDB and miRanda resources. The miRDB returned a list of 105 human miRNAs with a Target Score > 50, while the miRanda returned 918 miRNAs (out of 2656 input sequences, extracted from miRBase, see sheet_guide in Supplementary Table S2, sheet_miRDB and sheet_miRanda). All these miRNAs aligned with the investigated RNA. Seventeen miRNAs were shared by the two tools (i.e., miRDB output and the Top100 scoring sequences obtained with miRanda, details in Supplementary Table S2 sheet_comparison). Seven out of 17 miRanda/miRDB molecules are reported in the miRCancer dataset: i.e., hsa-let-7b-5p, hsa-let-7g-5p, hsa-miR-510-5p, hsa-miR-548p, hsa-miR-98-5p, hsa-miR-4500, hsa-miR-1283 (Supplementary Table S2, sheet_comparison). Regarding the sole miRDB output, 11 miRNAs are reported as cancer-associated according to the miRCancer resource, while 10 miRNAs predicted from miRanda with a high alignment score are reported within miRCancer.

About mRNA-mRNA interaction analysis, we found that 3 putative mRNAs can interact with MRSP31P5 transcript: MDM4 (at 3′UTR level), PCNXL3 and THBD (exon-exon interaction) (Supplementary Table S2, sheet_comparison and sheet_miRNA_miRCancer), according to public PARIS transcriptome-wide experiments [28] in RISE database.

**Figure 3:**
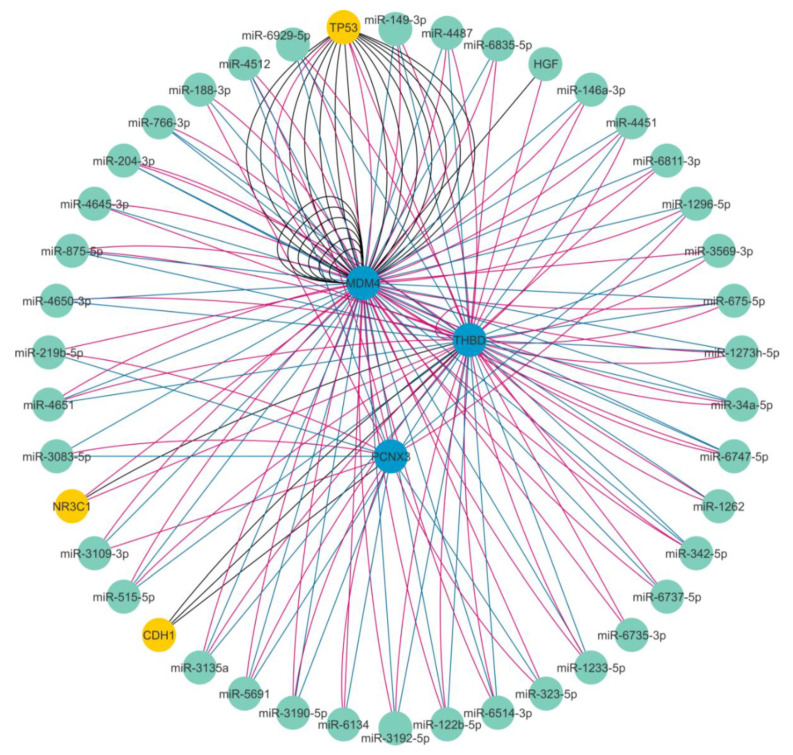
Overview of the ceRNA network showing the predicted interaction among mRNAs and miRNAs identified through in-silico analyses. The miRNAs are shown as green nodes, the mRNAs interacting with lncMRPS31P5 are shown as blue nodes, and the other mRNAs are shown as yellow nodes.

The expression levels of the MRPS31P5, MDM4, and THBD were investigated in our whole RNA-Seq datasets and we found that MRPS31P5, MDM4, and THBD were expressed at significantly higher levels in tumors than in normal mucosa (FC = 3.3 *p* = 0.0003; FC = 1.5 *p* = 0.004, FC = 2.6 *p* = 0.002, respectively), while the expression of PCNXL3 gene did not differ. Figure 3 and Supplementary Table S3 show the predicted interaction among these genes and miRNAs identified through these in-silico analyses.

## 3. Discussion

CRC has been postulated to start as a premalignant lesion, which, in association with several molecular events, gradually develops into cancer [29]. Genetic mechanisms are involved as shown by studies of twins indicate that up to 35% of all CRCs have a genetic component. Moreover, several hereditary CRC syndromes, such as hereditary non-polyposis colorectal cancer (HPNCC), familial adenomatous polyposis (FAP), MYH-associated polyposis (MAP), and the hamartomatous polyposis syndromes (HPS: Peutz–Jeghers, juvenile polyposis, and Cowden disease) have been identified and specific mutations in some genes, such as DNA mismatch repair genes, proofreading-impaired genes, adenomatous polyposis coli genes, etc. have been also detected [30]. Nevertheless, previous syndromes account only for less than 10% of all CRC cases. Therefore, our understanding of genetic risk factors for CRC is still incomplete and further studies are needed to find new distinct genetic components with specific biomarkers in non-hereditary, “sporadic” CRC.

Cancer biomarkers are increasingly used in clinical practice to identify early-stage cancers, evaluate the severity of oncological diseases, and predict the response to specific targeted therapies. The search for new cancer biomarkers (including epigenetic modifications, as well as the role of lncRNAs as epigenetic regulators) has been dramatically accelerated in the last years with the development of NGS technology and advancements in bioinformatics tools. Specifically, these innovative methods have been used in the present study to analyze the transcriptome of synchronous adenomatous and tumour tissues retrieved from sporadic CRC patients, and to identify structural rearrangements, i.e., fusion or chimera genes, which could be distinctive of the different evolutive events of the colorectal tumorigenesis.

In our study, we found, in about a third of patients, a fusion named MRPS31-SUGT1 generated through an intra-chromosomal translocation on chromosome 13. RT-PCR analysis showed that it was a tumor-specific event since it was detected in both adenoma and in tumor specimens, but not in normal paired tissues. Notably, Sanger sequencing and BLAST analyses among the amplicons produced from cDNA of polyps and carcinomas and MRPS31P5 showed higher sequence identity (100% similarity) than the fusion MRPS31-SUGT1 (90% similarity with exon 6 of MRPS31 and 98% similarity with exon 3 of SUGT1). These data are in agreement with those recently published by Wu and colleagues [27]. Even if the aim of their work was the study of chimeric RNAs as functional precursors of genes, the authors identified a chimeric gene, HNRNPA1L2-SUGT1 (H-S), whose sequence is highly similar to that of the ‘pseudogene’ MRPS31P5. This finding supports the hypothesis that MRPS31P5 is not actually a pseudogene of MRPS31, but rather a likely functional descendent of H-S chimera with implications in apoptosis and cell-cycle pathways. More importantly, these authors considered that MRPS31P5 could instead be transcribed on the basis of the sequence analysis. Very recently, the same authors have suggested that frequent chimeric RNAs are present in CRCs by studying The Cancer Genome Atlas (TCGA) CRC RNA-Seq datasets. Among others, they identified HNRNPA1L2-SUGT1 chimeric RNA in CRC patients and, in addition, it resulted significantly more abundant in two of six colon cancer cell lines as compared to normal colon cell line [31].

To study the role of the mRNA molecules in colorectal carcinogenesis, we performed both differential gene expression and functional enrichment analyses by means the Pan-Cancer Panel. This proved a significant functional enrichment in 1385 genes implicated in cancer and/or involved in fusion events. Of them, 329 were deregulated genes (97 down-regulated and 232 up-regulated) in tumors and 174 (three down-regulated and 171 up-regulated) in polyps when compared to normal paired tissues. A further analysis of functional enrichment in tumor and in synchronous adenoma samples identified the main categories enriched within up-regulated functions, including the cell cycle, cell development, cell growth, and proliferation, while those with inhibited functions were organic survival (death, morbidity, or mortality) and the apoptosis-related functions. These data from human CRC tissues are in accordance with those obtained from MRPS31P5 knockdown fibroblast HHF cells [27], and further stress the role of MRPS31P5 gene and the molecular pathway involved, principally cell cycle and apoptosis, also in sporadic CRC.

We performed several ancillary analyses based both on in-silico approaches implementation for evaluation of miRNA-mRNA interaction, and at the tissue level by using our RNA-Seq data for studying MRP31P5-mRNA interactions. We initially explored the functional role of these miRNAs by evaluating predicted miRNA-mRNA interaction by using miRDB and miRanda resources. As expected, since the MRP31P5 RNA is slightly expressed in colon tissue at a normal constitutional level, we did not retrieve a predicted or validated (and published) interactions between miRNAs and MRPS31P5. Afterward, we selected 17 miRNAs shared by the two tools that were predicted to target the MRPS31P5 mRNA. Finally, we ascertained whether these 17 miRNAs were present in the miRCancer database. Of the seven retrieved miRNAs, we hypothesized that the hsa-miR-4500 could be a player in CRC development, as it was found up-regulated in CRC cells induced apoptosis by means of ursoic acid [32]. A further miRNA likely involved in CRC was let-7b-5p, as it is shown implicated in modulating the TGFBR1 expression, via miRNA-mRNA binding site resulting in cancer-promoting consequences [33].

Subsequently, our top score miRNAs predicted to recognize sequence sites of the MRPS31P5 gene by means miRDB and miRanda resources, were analyzed by miRCancer tool. This query pinpointed a number of miRNAs-mRNA association indicating a possible role of MRPS31P5 gene as “miRNA sponge”. For example, the miR-30a-3p aligned with a high score according to the miRanda tool (Supplementary Table S2) and is reported as significantly down-regulated within CRC (miRCancer database). It is tempting to hypothesize that higher expression of MRPS31P5 could decrease the dosage of free-acting miR-30a-3p, thus deregulating miRNA target genes. Another example is given by the miR-1275, predicted from miRDB with a high target score and reported within miRCancer. Several reports showed that miR-1275 might function as an oncogene or tumor suppressor in various cancers included in CRC progression [34].

The interaction study among MRPS31P5-mRNAs molecules has provided further evidence to support the role of MRPS31P5 as a lncRNA molecule.

We highlighted three mRNA pairs, MRPS31P5/MDM4, MRPS31P5/PCNXL3, and MRPS31P5/THBD from RISE database. Interestingly, our RNA-Seq data showed that MRPS31P5, MDM4 and THBD were expressed at significantly higher levels only in cancer tissues, while PCNXL3 did not result to be differentially expressed. In agreement, MDM4 gene has been detected in about 50% of CRC patients and plays an important negative regulator role of p53 [35]. In CRC patients, MDM4 is regulated by several factors [36] and represents a target of miR-370 [37]. Regarding THBD gene, it encodes for thrombomodulinand which is resulted hypermethylated in early-stage CRC [38].

The results of ancillary analyses, those MRP31P5-mRNA obtained using our RNA-Seq data and those MRP31P5-miRNAs achieved using in-silico dataset, were integrated by means of lncRNA-miRNA-mRNA ceRNA networks (Figure 3): the analysis revealed several miRNAs on the constructed landscape network. These miRNAs indirectly interact with lncMRPS31P5 through a direct connection with MDM4 and THBD. An example is given by the miR-1273h-5p which presents a direct bridge joint with THBD and MDM4. The miR-1273h-5p is predicted by miRanda with a high score and is included among the seventeen miRNAs shared by both the miRDB and miRanda tools (Supplementary Table S2, sheet_comparison). The miR-1273 family has binding sites on several target gene mRNAs [39], including MDM4, but its biological role in CRC is unknown.

Although the mechanisms underlying the role of MRPS31P5 lncRNA as a “miRNA sponge” or as a mRNA regulator are unclear and still under investigation, these data confirm that lncMRPS31P5 may be an important gene expression regulatory factor, by means of the synergistic effects of lncRNA on mRNAs and miRNAs. The lncMPRS31P5 could take part in a ceRNA network by acting as a miRNA sponges, or/and it could be involved in tumorigenesis interacting with other mRNAs (i.e., MDM4, and THBD) and a number of predicted miRNAs (hsa-miR-4500, let-7b-5p, miR-1273h-5p). Over the past few years, epigenetic regulators and principally lncRNAs have emerged as biomarkers in several cancers including CRC. Most of the CRC related lncRNAs are upregulated and seem to function as miRNA “sponges” modulating both miRNA expression and function by competing for miRNA binding sites with endogenous mRNAs [10,21,22,23].

Although the low number of enrolled patients does not afford us to draw firm conclusions, the power and the main interest of our study is in the whole-transcriptome sequencing of eleven sporadic CRC patients with synchronous adenomas and carcinomas. In fact, we were able to identify MPRS31P5 as a novel genetic lncRNA marker that is already present in the early stage of colorectal carcinogenesis. The lncMPRS31P5 has been detected in synchronous lesions of three subjects having sporadic CRC, a finding which was corroborated by data of differential expression analysis, function enrichment analysis at tissue level, and in-silico integrated approaches. In our cohort of patients with synchronous lesions, no significant correlation was found among lncMRPS31P5 and each of the clinic-pathologic parameter analyzed (gender, age, localization, tumor stage, lymph node metastasis, and five-year survival) (Supplementary Table S4).

In conclusion, in patients with CRC and synchronous adenomas, lncMPRS31P5 gene represents a newly unreported and recurrent event and should be further investigated as a potential biomarker in CRC in a wider cohort of patient.

## 4. Materials and Methods

### 4.1. Clinical Samples and RNA Extraction

Eleven CRC patients (8 males, 3 females; mean age: 67 ± 12 years) with endoscopically detected synchronous adenomas and carcinomas were prospectively recruited from 2010 to 2013 at the Fondazione IRCCS-Casa Sollievo della Sofferenza Hospital Italy (Supplementary Table S4). All recruited patients were sporadic cases without family history of CRC. The study was conducted in accordance with the Declaration of Helsinki, and the protocol was approved by the Ethics Committee of the Fondazione IRCCS-Casa Sollievo della Sofferenza Hospital (Prot. N.132 CE/2015) and informed consent was signed by all patients. All experiments were performed in accordance with relevant guidelines and regulations.

Bioptic tissue from the adenomas and cancerous lesions, as well as from adjacent non-tumorous mucosa, were collected, and specimens immediately frozen in liquid nitrogen and stored at −80 °C.

Total RNA was extracted from the fresh frozen tissue samples using TRIzol reagent (Thermo Fisher Scientific, Somerset, NJ 08873, USA) and gentleMACS Dissociator (Miltenyi Biotec, Bergisch-Gladbach, Germany). The RNA purification was performed with RNeasy Mini Kit (Qiagen, Valencia, CA, USA) according to manufacturers’ instructions, and treated with DNase I RNAse free kit (Qiagen, Valencia, CA, USA) to remove genomic DNA. RNA quality was evaluated on the BioAnalyzer 2100 microcapillary electrophoresis system (Agilent Technologies, Palo Alto, CA, USA), and RNAs with an RNA integrity number (RIN) ≥7.0 were retained for the subsequent RNA-Seq analysis.

### 4.2. Whole Transcriptome Sequencing (RNA-Seq)

An aliquot (Ci = 100 ng/µL) of total RNAs was used to construct cDNA libraries according to the TruSeq Stranded Total RNA Sample Preparation kit, as provided by the manufacturer (Illumina, San Diego, CA, USA). In the first step, cytoplasmic and mitochondrial ribosomal RNAs (rRNAs) were removed using biotinylated, target-specific oligos combined with Ribo-Zero rRNA removal beads (Human Ribo-Zero Gold kit). Subsequently, the RNAs were purified, fragmented and a double-stranded cDNAs synthesized. The 3′-end adenylation and the adapters ligation of these cDNA fragments were performed, preparing them for hybridization onto a flow cell. The products were purified and enriched with PCR to create the final cDNA library.

To validate the quality and to assess the size distribution of cDNA library, an aliquot was loaded onto an Agilent High Sensitivity DNA chip and was running on Agilent Technologies 2100 Bioanalyzer (Agilent Technologies, Santa Clara, CA, USA). For the accurate quantitation of the DNA library, the samples were analyzed by using a fluorometric based system (Qubit dsDNA HS Assay System; Thermo Fisher Scientific, Somerset, NJ, USA). The DNA libraries were pooled and an aliquot loaded into a High Output flow cell and sequenced through NextSeq500 System (Illumina, San Diego, CA, USA). Considering the potential of RNA-Seq and its versatility, a paired-end approach (2 × 75 bp) was used with around 80 million reads per sample.

### 4.3. Fusion Detection

RNA-Seq data were analyzed in order to discover the potential fusion genes in the different tumor stages. Raw data (.fastq files) were quality-controlled using the FastQC v0.11.5 software package (http://www.bioinformatics.babraham.ac.uk/projects/fastqc/). Reads were discarded if the average per-base phred values resulted in less than 20, or trimmed by Trimmomatic [40] if the phred values of more than 5% of nucleotides at the extremities of the reads were lower than 20. Residual adapter sequences were removed by cutadapt [41]. Residual reads were analyzed with two tools, Chimerascan [42] and EricScript [43], on a list of 1880 genes and genomic regions, searched for with the following keywords: “colorectal cancer”, “colorectal sporadic” “Lynch syndrome” and “familial polyposis” in the NCBI gene database http://www.ncbi.nlm.nih.gov/gene/; accessed on 15 September 2016) (Supplementary Table S1). Each software package was run with standard parameters, yielding a list of putative fusion genes, annotated with the coordinates of the portions of the partner genes, together with the estimated breakpoints, the type of fusions (e.g., inter-chromosomal, read through), and a reliability score. Their results were pooled and considered together for further validation.

### 4.4. Fusion Gene Validation by RT-PCR and Sanger Sequencing

The expression of the candidate fusion genes was validated by RT-PCR. A mixture containing 0.1 μg of total RNA from each sample was reverse transcribed for 10 min at 25 °C, and 2 h at 37 °C using the High Capacity cDNA Reverse Transcription Kit (Thermo Fisher Scientific, Somerset, NJ, USA). Junction PCR specific primers were designed based on the RNA-Seq chimeric junction reads using primer3 software (https://primer3plus.com/primer3web/primer3web_input.htm) and the amplicon sequences were checked by BLAST against the human genome to ensure specificity.

PCR was performed in a final volume of 25 μL containing 2.5 μL 10× PCR Buffer (Thermo Fisher Scientific, Somerset, NJ, USA), 2.5 mM dNTPs, 25 mM MgCl2, 15 pM junction specific PCR primers, 0.15 μL AmpliTaq Gold polymerase (Thermo Fisher Scientific, Somerset, NJ, USA), and 1 μL cDNA. Cycling PCR conditions consisted of an initial 10 min denaturation step at 94 °C, followed by 35 cycles of 94 °C for 1 min, 58 °C for 1 min and 72 °C for 1 min, with a final extension at 72 °C for 10 min. PCR products were visualized by ethidium bromide staining on 3% agarose gels.

cDNA samples from matching normal tissue were used as controls to be able to confirm that fusion genes were tumor-specific.

The junction sequences of potential fusion genes, PCR primers sequence, size of PCR products will be available upon request. The amplicons were sequenced from both ends using an aliquot (3.2 pM) of the PCR reaction primers in presence of BigDye Terminator Cycle Sequencing Kit v. 1.1 (Thermo Fisher Scientific, Somerset, NJ, USA). After purification by using centrisep columns (Thermo Fisher Scientific, Somerset, NJ, USA), sequencing reactions were loaded on 3500 DX Genetic Analyzer capillaries (Applied Biosystems, Foster City, CA, USA) and analyzed using the Sequencing Analysis software v5.4.

### 4.5. Fusion Gene Validation and Functional Enrichment Analysis by Targeted RNA-Seq

An aliquot of total RNA extracted from the eleven CRC patients was used for constructing cDNA libraries with TruSight RNA Pan-Cancer Panel Kit (Illumina, San Diego, CA, USA) following the manufacturer’s protocol. The cDNA libraries were enriched for 1385 genes implicated in cancer and/or involved in fusion events. Briefly, double-stranded cDNA was generated from RNA fragments using random primers by means of a first and second-strand synthesis. The 3′ end adenylation and the ligation of sequencing adapters to the cDNA fragments were carried out. Finally, the enriched library was created performing PCR amplification of cDNA fragments and hybridization of sequence-specific probes to the coding regions of the expressed cancer-associated genes. The enriched library quality was tested both on Agilent Technologies 2200 Bioanalyzer using D1000 ScreenTape Assays (Agilent, Santa Clara, CA, USA), and on Qubit dsDNA HS Assay System (Thermo Fisher Scientific, Somerset, NJ, USA). Paired-end RNA-sequencing was performed on a NextSeq500 system (Illumina, San Diego, CA, USA) and raw sequencing data were demultiplexed to fastq files for downstream analysis.

Targeted RNA-Seq data were further used to infer differential gene expression when comparing three matched groups of samples: tumors, polyps, and normal mucosa. To this aim, we aligned and counted all piled-up reads by STAR ver. 2.7 [44]. Normalized counts were used to perform a PCA and thus check the correct assignment of samples to their groups. Then, differentially expressed genes were obtained using DESeq2 ver. 1.26 [45]. Genes with adjusted *p*-values ≤ 0.05 (by the Benjamini–Hochberg procedure) and fold-change values exceeding ±1.5 were selected for in-silico functional enrichment analysis. It was conducted using Ingenuity Pathway Analysis (IPA, QIAGEN, Hilden, Germany) and by querying the Gene Ontology (GO). The entire procedure was based on the prior calculation of the activation z-scores, which were used to infer the activation states of functions. Z-scores > 2 and <−2 were assigned to biological functions that were predicted to be functionally active or inhibited, respectively.

### 4.6. Competing Endogenous RNA Analysis

The possible functional roles of the MRPS31P5 gene was initially investigated by first searching for precomputed miRNA-mRNA interactions in specialized miRNA databases. Second, two alternative alignment strategies were implemented to detect significant interacting miRNAs initially used the miRDB resource [46] (http://mirdb.org/custom.html, “Submission Type > mRNA Target Sequence”) and the reference FASTA sequence of the investigated RNA molecule in order to obtain a ranked list of paired human miRNAs. Secondarily, we ran the miRanda software [47] with the following input data: RNA FASTA sequence; multi-FASTA file containing all mature miRNA sequences from miRBase [48] (ftp://mirbase.org/pub/mirbase/CURRENT/, “mature.fa.gz”). A consensus list of potentially paired miRNAs was obtained by comparing miRDB results with the Top100 scoring miRNAs from miRanda output. We also considered public collections of cancer-associated miRNAs to evaluate the pathophysiological role of pairing miRNAs and, ultimately, of the competing chimeric RNA. We implemented the miRCancer database (downloaded from http://mircancer.ecu.edu/download.jsp, “miRCancerOctober2019.txt” file) [49], because it is frequently updated and easy to manage.

### 4.7. Study of RNA-RNA and RNA-Protein Interactions

Interactions among MRPS31P5 and RNA molecules were also explored by querying the RISE database (http://rise.life.tsinghua.edu.cn/index.html). This resource collects data from transcriptomic NGS or more targeted experimental approaches, that particularly focalize on RNA-RNA interactions (e.g., PARIS, LIGR-seq, RAP-RNA.). For each querying gene, genomic positions, functional annotations, interacting genes, involved cell line/tissue, experimental techniques, and Pubmed accessions are possibly returned. Evidence for RNA binding protein (“RBP”) interactions, RNA editing-modification events, and expression levels are also given for matching genes. Furthermore, an RNA–protein to protein interaction network was built using IPA which reported relationships like co-expression, activation/inhibition, co-localization, phosphorylation, regulation of binding or transcription, RNA–RNA interactions, and others as edges and molecules as nodes of a network. The resulting network is a multi-graph, technically speaking since multiple edges are allowed between any couple of molecules, meaning that any two molecules can be linked by more than one evidence of interaction. The network was drawn using Cytoscape 3.7.

## Figures and Tables

**Table 1:** List of putative fusion genes selected by EricScript (white rows) and ChimeraScan (grey rows) tools, with the name of the partner genes, the estimated breakpoints, the type of fusions (e.g., Inter-chromosomal, Read Through) and a reliability score. + = sense or coding strand; - = antisense or not coding strand

Fusion	Gene Name 5p	Gene Name 3p	chr 5p	Breakpoint 1 (End 5p)	Strand 5p	chr 3p	Breakpoint 2 (Start 3p)	strand 3p	Fusiozn Type	JunctionSequence	Score
**RNF123-STAT3**	RNF123	STAT3	3	49,728,680	+	17	49,728,680	-	inter-chr	ccgcaagagctataggctgacctcagatgctgagaaatccagggtcacagCTACTCGGGAGGCTGAGGCAGGAGAATCGCTTGAACCTGAGAGGCGGAGG	0.91586235
**PLK1-ERN2**	PLK1	ERN2	16	23,701,614	+	16	23,702,074	-	Cis	gtgggttctacagccttgtccccctccccctcaaccccaccatatgaattGCTGGGTGCAGTGGCTCACACCTGTAATCCCAGCATTTTGGGAGGCTGAG	0.690112004
**MRPS31-SUGT1**	MRPS31	SUGT1	13	41,323,274	-	13	53,231,667	+	intra-chr	gtggacaaaagaggggaaactatgggagttcccaattaacaatgaagcagGAGCTGACTAAGGCTTTGGAACAGAAACCAGATGATGCACAGTATTATTG	0.734629203
**LPHN1-SUZ12**	LPHN1	SUZ12	19	14,316,797	-	17	30,267,305	+	inter-chr	cgagccgcaggagagacacgctgggccgaccccagagaggcgctggacagAGCCAACACAGATCTATAGATTTCTTCGAACTCGGAATCTCATAGCACCA	0.855475528
**EIF5AL1-MSH3**	EIF5AL1	MSH3	10	81,274,508	+	5	81,274,508	+	inter-chr	aagactgtgaaaatgaatccagaggtgacccaagcattgaatttaacaatGGTGGCTCATGCCTGTAATCCCAGCACTTTGGGAGGCCAAGGTAGGCAGA	0.532811583
**GUCY2C-PLBD1**	GUCY2C	PLBD1	12	14,765,813	-	12	14,721,126	-	Read- Through	accttccactctggaaccttattccagcagttgttccagggagcttctacCTGTGGAGGCCTCTCCAGAAACAGCAGAGGATCCGAGCTGCGTGTAGGCA	0.896360711
**HSPE1-MOB4**	HSPE1	MOB4	2	198,367,852	+	2	198,388,348	+	Read- Through	aagttcttctcccagaatatggaggcaccaaagtagttctagatgacaagGATTTCTATAATTGGCCTGATGAATCCTTTGATGAAATGGACAGTACACT	0.821493951
**PDLIM2-CCAR2**	PDLIM2	CCAR2	8	22,455,537	+	8	22,463,248	+	intra-chr	agagattggctgtgggcctcagtttccccattttataaagttttaaaatctGCCTTTTCCCCACGACTCTGAAAGAGGACAGCGTTCCCAATGTCCCAGTTT	5
**HPSE2-HSD11B2**	HPSE2	HSD11B2	10	100,995,631	-	16	67,469,859	+	inter-chr	tctcttcctactgggtctcgctagtgactaattgtccttatctaaagtgtgGGCCTGTGGGGCCTCGTCAACAACGCAGGCCACAATGAAGTAGTTGCTGAT	2
**HDAC1-MARCKSL1**	HDAC1	MARCKSL1	1	32,799,223	+	1	32,799,429	-	Adjacent_ Converging	agatactattttcatttttgtgagcctctttgtaataaaatggtacatttcTAAAGCACCACTAAAGGGACGACATTTATTCCTTTTCCAAATGTTACAGTA	2
**ARSA-TNS4**	ARSA	TNS4	22	51,066,600	-	17	38,632,079	-	inter-chr	gccggtaccgggctgcgggcgcttccgcctcggccccgccccgtgacctgtCTTACTGTTTTGCAAAGACAAACATTTTATTTTTCATGATAGGAGCTGTAG	4
**ERBB2-MIEN1**	ERBB2	MIEN1	17	37,883,255	+	17	37,885,408	-	Adjacent_ Converging	cccgggcgctgggggcatggtccaccacaggcaccgcagctcatctaccagATTAGTGTTTGTAGCGCCACTTTACTGCCAATAGCTGACATTGCCCTGGGT	4

## Supplementary Materials

Supplementary materials can be found at https://www.mdpi.com/1422-0067/21/19/7120/s1. Figure S1. (UCSC Genome Browser) LncMRPS31P5 is located in a region prone to rearrangements, on chromosome 13. A multitude of small duplicated sequences are annotated in the MRSP31P5 surroundings that could favor intra-chromosomal or extra-chromosomal non-homologous recombination events. This region is conserved from human to Rhesus genomes, while is lost at higher phylogenetic distances (Marmoset genome). Figure S2. Principal component analysis (PCA) of Pan Cancer panel data. The points are colored by group status: red represent normal samples, blue represent polyp samples and green represent tumor samples. PCA showed a similar distribution of samples among different groups (normal, polyp and tumor tissues). Tables S1–S3. List of predicted molecular interactions between mRNAs and miRNAs identified through in-silico analyses. Table S4. The association of lncMRPS31P5 with clinical parameters. TNM=lymph node metastasis. All comparisons were not statistically significant (*p* > 0.05).
