# Supplementary material for: Transcriptome and Gene Fusion Analysis of Synchronous Lesions Reveals lncMRPS31P5 as a Novel Transcript Involved in Colorectal Cancer
Source: Int J Mol Sci. 2020 Sep 27;21(19):7120. doi: 10.3390/ijms21197120 (PMC7582694; doi:10.3390/ijms21197120)

**Supplementary Figure 1.** (UCSC Genome Browser) LncMRPS31P5 is located in a region prone to rearrangements, on chromosome 13. A multitude of small duplicated sequences are annotated in the MRSP31P5 surroundings that could favor intra-chromosomal or extra-chromosomal non-homologous recombination events. This region is conserved from human to Rhesus genomes, while is lost at higher phylogenetic distances (Marmoset genome).


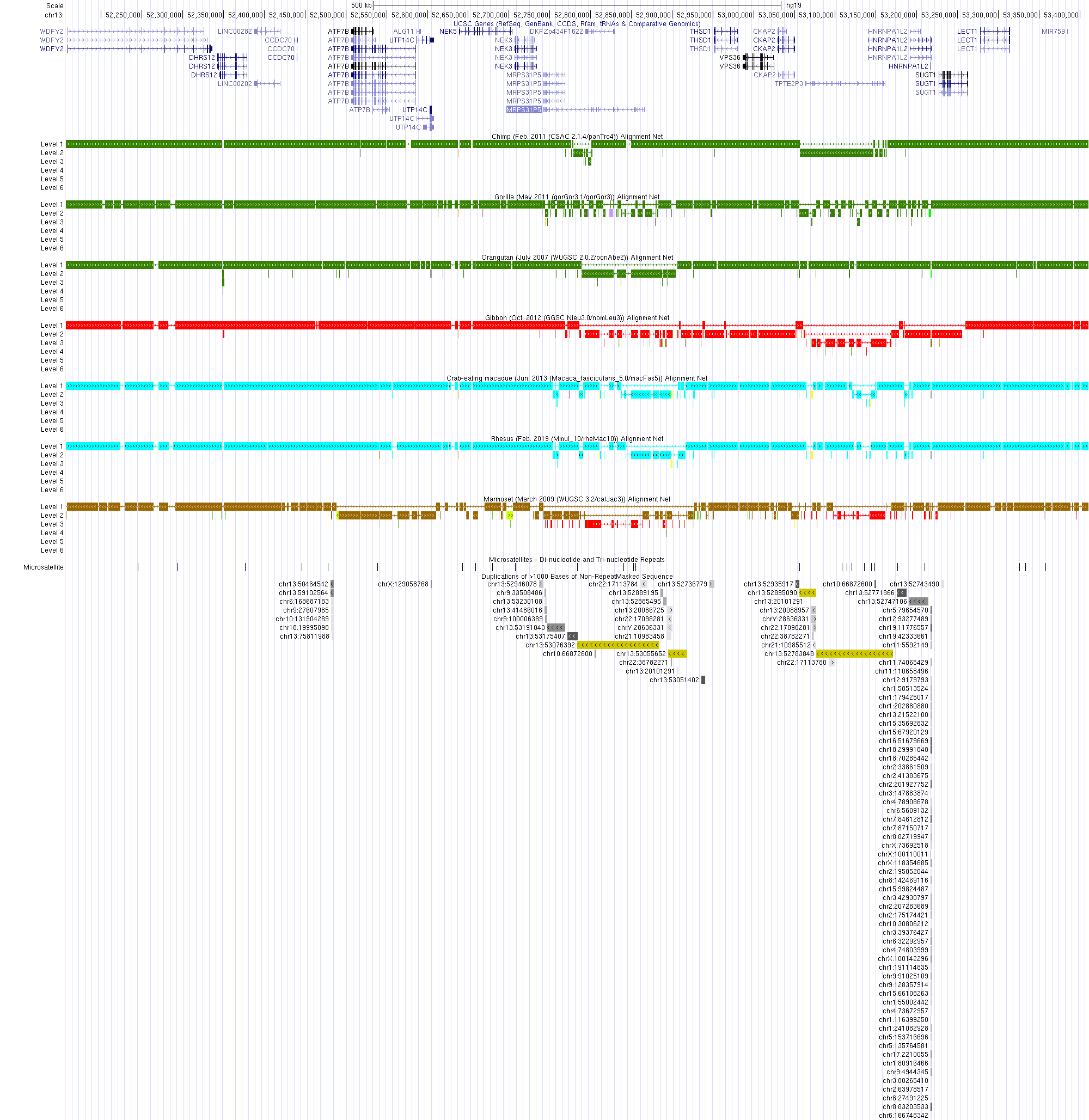

Supplement: Supplementary file 1 [file ijms-21-07120-s001.zip › Supplementary Figure 1.docx]
