# Supplementary material for: Transcriptome and Gene Fusion Analysis of Synchronous Lesions Reveals lncMRPS31P5 as a Novel Transcript Involved in Colorectal Cancer
Source: Int J Mol Sci. 2020 Sep 27;21(19):7120. doi: 10.3390/ijms21197120 (PMC7582694; doi:10.3390/ijms21197120)

**Supplementary Figure 2.** Principal component analysis (PCA) of Pan Cancer panel data. The points are colored by group status: red represent normal samples, blue represent polyp samples and green represent tumor samples. PCA showed a similar distribution of samples among different groups (normal, polyp and tumor tissues).


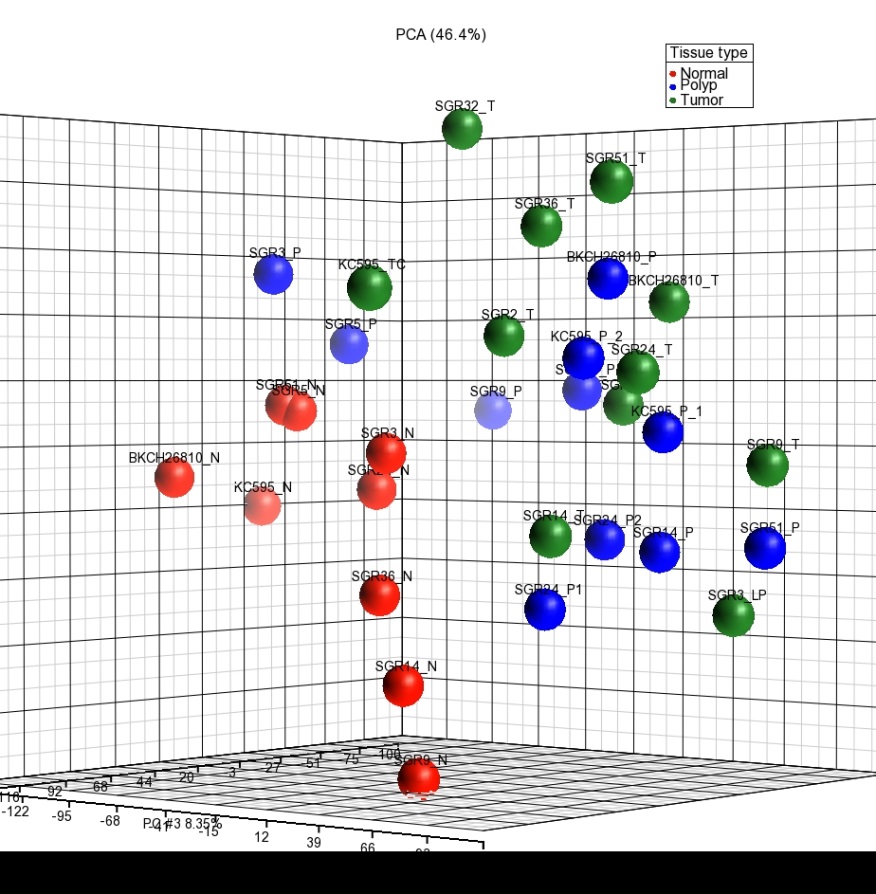

Supplement: Supplementary file 1 [file ijms-21-07120-s001.zip › Supplementary Figure 2.docx]
