# Supplementary material for: Transcriptome and Gene Fusion Analysis of Synchronous Lesions Reveals lncMRPS31P5 as a Novel Transcript Involved in Colorectal Cancer
Source: Int J Mol Sci. 2020 Sep 27;21(19):7120. doi: 10.3390/ijms21197120 (PMC7582694; doi:10.3390/ijms21197120)
Supplement: Supplementary file 1 [file ijms-21-07120-s001.zip › supplementary table 4.docx]

|  | **lncMRPS31P5_positive** | **lncMRPS31P5_negative** | **p-value** |
| --- | --- | --- | --- |
| **Gender** | | | |
| **M (8)** | 2 | 6 | ns |
| **F (3)** | 1 | 2 |  |
|  |  |  |  |
| **Age of onset** | | | |
| **<50 years** | 0 | 0 | ns |
| **>50 years** | 3 | 8 |  |
|  |  |  |  |
| **Localization** | | | |
| **Proximal (4)** | 0 | 3 | ns |
| **Distal (7)** | 3 | 5 |  |
|  |  |  |  |
| **Dukes stage** | | | |
| **A** | 0 | 0 | ns |
| **B** | 1 | 3 |  |
| **C** | 2 | 5 |  |
| **D** | 0 | 0 |  |
|  |  |  |  |
| **TNM stage** | | | |
| **I-II** | 1 | 3 | ns |
| **III-IV** | 2 | 5 |  |
|  |  |  |  |
| **N0** | 1 | 3 | ns |
| **N1-2** | 2 | 5 |  |
|  |  |  |  |
| **Survival 5 years** | | | |
| **Yes** | 1 | 5 | ns |
| **No** | 2 | 3 |  |

**Supplementary Table 4**. The association of lncMRPS31P5 with clinical parameters. TNM=lymph node metastasis. All comparison were no statistically significant (p>0.05).

| Pts | MRPS31-SUGT1 | Gender | Age of onset | Localization | Dukes stage | TNM stage | N stage | G | 5y survive |
| --- | --- | --- | --- | --- | --- | --- | --- | --- | --- |
| Maresca Eligio | Pos | M | 63 | Distal | B | T3N0M0 (IIa) | N0 | 2 | Y |
| Siminelli Michele | Pos | M | 83 | Distal | C | T3N1M0 (III) | N1 | 2 | N |
| Tigrano Arcangela | Pos | F | 89 | Distal | C | T3N1M0 (IIIB) | N1 | 2 | Y |
| Donatacci Rosa | Neg | F | 64 | Proximal | C | T3N1M0 (IIIB) | N1 | 2 | Y |
| Palladino Maria Pia | Neg | F | 68 | Distal | B | T2N0M0 (I) | N0 | 1 | Y |
| Urgo Angelo | Neg | M | 66 | Proximal | B | T3N0M0 (IIA) | N0 | 2 | Y |
| Rispoli Matteo | Neg | M | 52 | Proximal | B | T3N0M0 (IIA) | N0 | 2 | Y |
| Zilli Giuseppe | Neg | M | 73 | Distal | C | T3N1M0 (IIIB) | N1 | 3 | N |
| Meola Antonio | Neg | M | 54 | Distal | C | T3N1M0 (IIIB) | N1 | 2 | N |
| Viceconti Federico | Neg | M | 66 | Distal | C | T3N1M1 (IV) | N1 | 2 | N |
| Morfeo Giancarlo | Neg | M | 51 | Distal | C | T3N1M0 (IIIB) | N1 | 1 | Y |
