# Supplementary material for: Transcriptome and Gene Fusion Analysis of Synchronous Lesions Reveals lncMRPS31P5 as a Novel Transcript Involved in Colorectal Cancer
Source: Int J Mol Sci. 2020 Sep 27;21(19):7120. doi: 10.3390/ijms21197120 (PMC7582694; doi:10.3390/ijms21197120)
Supplement: Supplementary file 1 [file ijms-21-07120-s001.zip › Supplementary Table 1_Panza A et al.pdf]

Gene Symbol (NCBI gene database)					
**colorectal cancer_ncbi**	**colorectal cancer sporadic_ncbi**	**Lynch_ncbi**	**crc familial**	**poliposys**	**our data resequencing**
TGFB1	BRCA2	CYP17A1	HPP1	APC2	CTNNA1
TP53	IFNG	RNASEL	SCN1A	TMEFF2	NF1
CDH1	MTOR	MAX	HNF4A	AMER1	RPS20
TNF	JAK2	KAT2A	EGLN1	APCDD1	SDHA
SMAD7	SERPINE1	PTPRG	BMPR2	REEP5	SDHC
MYC	PON1	ANXA10	DES	SPATA13	SDHD
EGFR	CCR5	RINT1	BDNF	REEP6	
VEGFA	F5	LRRFIP2	TNFRSF11B	APCDD1L	
CDKN1A	septin 9		RAB27A	HLA-DRB1	
MTHFR			DACH1	CFTR	
IL6			TCF12	HLA-B	
APOE			CYP2C19	HLA-A	
ERBB2			TP63	HLA-DQB1	
HIF1A			CRYAB	ADRB2	
NFKB1			HMGA2	HLA-C	
ADIPOQ			PLA2G2A	IL1A	
ABCB1			ID2	HLA-DQA1	
MMP9			SORT1	IL33	
PTGS2			PROX1	PTX3	
IL10			CARD8	MUC5AC	
CRP			AKAP9	TSLP	
CDKN2A			APBA1	AXIN1	
AKT1				MMP8	
COLCA1				BTRC	
ESR1				CCL11	
VDR				PDE4D	

BRAF				HLA-DRA	
PPARG				MUC5B	
KRAS				CCR3	
TLR4				CSNK2A2	
CXCL8				TNFSF4	
STAT3				IL9	
PTEN				COL11A1	
CTNNB1				FH	
DCC				CCR1	
TCF7L2				IGHE	
NOS3				SCRIB	
IL1B				DNAJA3	
GSTM1				CCL24	
IGF1				MT-CO2	
APP				DLG3	
BCL2				MAPRE2	
BRCA1				SCGB3A1	
CXCR4				USP15	
GATA3				IFRD1	
MDM2				MAPRE3	
COLCA2				DOCK4	
MMP2				MIR663A	
SLC6A4				DCBLD2	
GSTT1				PSG9	
MAPK1				FOCAD	
CCND1				SAPCD1	
AR				SCGB1C1	
BIRC5					
GSTP1					

TGFBR2					
COMT					
TERT					
LEP					
XRCC1					
CCL2					
HFE					
SOD1					
CTLA4					
FAS					
TLR2					
ATM					
KIT					
CXCL12					
ICAM1					
CD44					
ESR2					
PIK3CA					
IL17A					
HMOX1					
AGT					
MAPK14					
IL18					
SPP1					
RB1					
RELA					
NOTCH1					
IL4					
MET					

VWF					
NOD2					
ITGB3					
GJB2					
SRC					
FOXP3					
HLA-G					
RET					
CASP3					
PRNP					
CYP1A1					
MLH1					
SOD2					
ITGB1					
INS					
GSK3B					
CYP2C9					
NFE2L2					
IGF1R					
MAPK3					
SIRT1					
EDN1					
IL1RN					
AGER					
CDKN1B					
AGTR1					
HMGB1					
BAX					
CAV1					

JUN					
APC					
MMP1					
TNFSF10					
SP1					
PARP1					
RAC1					
MUC1					
MIR21					
ABCG2					
NOS2					
HGF					
KDR					
CD14					
ERCC2					
EP300					
UGT1A1					
PARK2					
FTO					
MGMT					
RHOA					
PTK2					
VHL					
PGR					
MAPK8					
HRAS					
NAT2					
MSH2					
FGF2					

MIF					
HSP90AA1					
EZH2					
CASP8					
F3					
NPM1					
LCN2					
STAT1					
IGFBP3					
MPO					
EGF					
LGALS3					
PTPN11					
FN1					
CCL5					
MKI67					
MMP3					
ERCC1					
HDAC1					
ALB					
CYP2E1					
FASLG					
INSR					
CYP3A5					
TP73					
OGG1					
IGF2					
LEPR					
FLT1					

E2F1					
IDH1					
ALK					
FGFR2					
TNFRSF1A					
BCL2L1					
ALDH2					
AURKA					
RETN					
CHEK2					
SMAD4					
TYMS					
GJA1					
APEX1					
CDK1					
BMP2					
FGFR3					
CD40					
PRKCD					
GNAS					
TIMP1					
SLC2A1					
PLAUR					
PLK1					
CTGF					
C3					
IL13					
CSNK2A1					
PLAU					

TLR3					
GRB2					
RAD51					
MICA					
PPARGC1A					
MCL1					
PDCD1					
CYP1B1					
CXCL10					
PROM1					
BSG					
CASR					
NQO1					
POU5F1					
HSPA5					
F7					
PCNA					
CNR1					
NAMPT					
APOBEC3G					
CD274					
SMAD3					
CLU					
GH1					
FGF23					
CBL					
CREB1					
MMP14					
IL2RA					

DPP4					
PIK3R1					
IRS1					
FGFR1					
HSPB1					
RASSF1					
MIR146A					
THBS1					
AHR					
HSPA4					
IL12B					
PML					
VCAM1					
XIAP					
FCGR2A					
ITGAV					
SHH					
CALCA					
SYK					
NFKBIA					
DNMT1					
HAMP					
EPO					
CDC42					
ITGB2					
CYP1A2					
ENG					
TWIST1					
NLRP3					

TGM2					
HNF1A					
B2M					
EZR					
TNFRSF1B					
PDGFRA					
BMI1					
S100B					
ADM					
FOXO3					
SELE					
SOX2					
CD209					
XRCC3					
IDO1					
IL15					
HP					
PRKDC					
VIM					
FOXO1					
MMP7					
VEGFC					
TOP2A					
ATP7B					
EGR1					
LGALS1					
MIR155					
FHIT					
CHI3L1					

NBN					
STK11					
ANGPT2					
TGFBR1					
HPSE					
FCGR3A					
F2R					
PDGFRB					
BCL2L11					
CHEK1					
SOCS3					
NOTCH3					
DKK1					
DICER1					
SHC1					
LRP1					
YAP1					
BMP4					
BECN1					
NRAS					
IL4R					
CEBPB					
EPAS1					
ABCC1					
ERBB3					
LTF					
CBS					
F2RL1					
PIK3CG					

IFNB1					
CD34					
ITGA2					
PAK1					
FOXM1					
HSPD1					
RUNX3					
MIR34A					
CAT					
POLG					
MME					
TIMP2					
SMAD2					
HDAC2					
PAX6					
ADAM17					
PTH					
ADH1B					
EPHX1					
SNAI1					
XPC					
SDC1					
CYP2A6					
IKBKB					
SPARC					
KLRK1					
SKP2					
S100A9					
IL23R					

FOS					
PRKCB					
MYD88					
FUS					
STIM1					
SERPINF1					
CFLAR					
FLNA					
TXN					
GDF15					
SHBG					
CD28					
CDX2					
MTR					
NME1					
AHSG					
SMARCB1					
SLCO1B1					
WRN					
CA9					
CASP9					
SOCS1					
CCNB1					
TNFAIP3					
S100A4					
EPCAM					
OLR1					
NR1I2					
TOP1					

ROCK1					
BCL6					
MUTYH					
DNMT3B					
YBX1					
PTPRC					
TPH2					
ABO					
PTPN1					
LIPC					
PPARD					
XPO1					
MSH6					
KL					
SOX9					
NF2					
GZMB					
CHGA					
BTK					
ABCC2					
PDGFB					
ALDH1A1					
GPX1					
BST2					
ITGA5					
RPS6KB1					
XRCC6					
IL12A					
NTRK1					

L1CAM					
CTSD					
NCOA3					
XRCC5					
CX3CR1					
ALOX5					
MIR145					
KLF4					
LYN					
PKD1					
CDKN2B					
ERBB4					
NTRK2					
PKM					
PLCG1					
PGF					
MAP2K1					
GC					
CD38					
NGFR					
NES					
HSD11B1					
TNFRSF10B					
SAA1					
IL22					
PRKAA1					
ID1					
ANXA1					
GLI1					

CXCR2					
STAT6					
PTCH1					
MUC16					
MMP13					
UCHL1					
HLA-E					
ILK					
MTRR					
ANGPT1					
IGFBP1					
CSF2					
SERPINB5					
STAT5A					
KISS1					
DNMT3A					
AICDA					
FASN					
PTGS1					
CRHR1					
MYB					
ZEB1					
TNC					
CD24					
HDAC3					
CCR7					
CEACAM1					
BMP7					
EPHA2					

IRF1					
INHBA					
CD55					
BLM					
PTTG1					
IDH2					
MTDH					
CXCR3					
HMGA1					
SPINK1					
JAK1					
PEBP1					
S100A8					
STAT5B					
FBXW7					
DPYD					
RARB					
GSN					
PXN					
JAG1					
HAVCR2					
CIITA					
KITLG					
PDPN					
IL23A					
CSF1					
CA2					
STMN1					
NANOG					

PPP2CA					
IGFBP2					
CTTN					
NRP1					
NAT1					
KRT19					
SPHK1					
ANG					
AKT2					
KRT18					
DCN					
STUB1					
CYR61					
STAT4					
FABP2					
AGTR2					
RAN					
CAPN10					
TGFA					
GATA4					
XBP1					
EWSR1					
CASP1					
CLOCK					
ITGA6					
NDRG1					
ITGB4					
POLB					
BDKRB2					

EDNRB					
DAPK1					
SNAI2					
TEK					
FLT4					
CD247					
FGFR4					
ERCC5					
SULT1A1					
CEACAM5					
ALOX15					
CTNND1					
ADD1					
ADH1C					
BAD					
CLDN1					
CTSL					
CD9					
EIF4EBP1					
TAC1					
KPNA2					
CDK6					
PDCD4					
KEAP1					
MTA1					
FOLH1					
XPA					
HSD11B2					
GSTA1					

DIABLO					
AXL					
PLA2G4A					
TACR1					
PF4					
TYMP					
RIPK1					
NR4A1					
TNFRSF10A					
IGF2R					
CXCL1					
CCL20					
CD86					
MIR126					
MRE11A					
IL27					
TFAP2A					
SDHB					
RASA1					
MAD2L1					
LOX					
SFN					
KDM1A					
CSK					
ATF3					
LCT					
HNRNPA1					
IRS2					
XRCC4					

ADIPOR1					
CD82					
TSC1					
LEF1					
TP53BP1					
CYLD					
BIRC3					
MFN2					
TLR5					
SFRP1					
SLC19A1					
MDK					
ITGA3					
CDH13					
CADM1					
PKD2					
HIST3H3					
MIR221					
TCF4					
TNFSF13					
GATA2					
CYP24A1					
MUC2					
HOTAIR					
SLC9A3R1					
HNRNPK					
TCF3					
ARF6					
FSCN1					

PRLR					
ZEB2					
WNT1					
NOTCH2					
IL32					
CSNK2B					
PITX2					
KLF6					
ABCC4					
HMGCR					
FEN1					
TNFSF12					
CD80					
ERCC4					
PDX1					
NR4A2					
MAP1LC3A					
KAT5					
CGA					
ANGPTL4					
RAB5A					
TUBB3					
COL4A5					
ARG1					
FANCD2					
MIR27A					
NCOA2					
ACKR3					
ZFP36					

BUB1B					
MAPK9					
S1PR1					
NEDD4L					
PTP4A3					
POU2F1					
CXCR1					
RAD50					
CDC20					
ARID1A					
APAF1					
TSPO					
BBC3					
TPM1					
ITLN1					
CXCL13					
ALCAM					
GAS6					
MMP12					
MIR200C					
TFPI2					
CASP7					
UGT2B7					
IL16					
MIR210					
ENPP2					
PMS2					
COPS5					
PSCA					

BAG1					
SATB1					
JUP					
MST1R					
MAP2K4					
UGT1A9					
ANPEP					
DLC1					
ARNT					
TNFRSF6B					
IL17F					
HSD17B1					
KLF5					
USP7					
PDIA3					
PTGER4					
UNG					
ALOX12					
ANO1					
CCR6					
MIR223					
MIR196A2					
MCM2					
HSPA9					
FGF7					
THPO					
FUT2					
MYOD1					
RGS2					

BNIP3					
LDHA					
RTN4					
NT5E					
GLUD1					
MECOM					
CCND2					
ATG16L1					
MAPK7					
PRDX2					
RFC1					
FOXP1					
SET					
MIR29A					
LGR5					
MIR17					
ESRRA					
BIRC7					
TFF3					
DDX3X					
RPSA					
HPGD					
IGFBP7					
FOLR1					
UHRF1					
WNT3A					
LAMC2					
TIAM1					
RECK					

AMACR					
MIR31					
EPHB2					
GATA6					
SLC3A2					
MAP2K2					
PTGES					
TTK					
GLI3					
PLD2					
SLCO1B3					
ZBTB16					
PLD1					
LIG4					
SF3B1					
PLCE1					
SLIT2					
FOXC2					
MIR203A					
DEK					
CLDN4					
PYCARD					
ITGA1					
GAL					
NDRG2					
ROCK2					
MIR143					
CCL21					
MIR205					

ST14					
EPHB4					
PRKAA2					
LY96					
PKLR					
MCM7					
FAP					
PTPA					
SLC22A4					
KIAA1524					
SULF1					
CD8A					
NTRK3					
GAST					
PTGER2					
PPM1D					
CXCL5					
MIRLET7A1					
HHEX					
BRIP1					
NR1H2					
DROSHA					
CDH3					
FADS2					
TMSB4X					
SNCG					
EHMT2					
ARHGDIA					
CYCS					

BMPR1A					
MIR137					
DKC1					
CCL18					
ING1					
EFEMP1					
FADS1					
TNFRSF9					
E2F3					
COX2					
TACSTD2					
MIR16-1					
WIF1					
HES1					
CD276					
AGR2					
TCN2					
FHL2					
NTN1					
MASP2					
LIN28A					
HOXB13					
RHOB					
LTC4S					
MIR499A					
CAMK2G					
CEACAM6					
EFNB2					
SLC5A1					

UBE2C					
TPP1					
ADIPOR2					
SERPINA12					
WNK1					
PER2					
XRCC2					
ODC1					
KLK6					
SOX4					
MALAT1					
UGT1A6					
TK1					
BGN					
TPSAB1					
CHFR					
PROZ					
MACC1					
CES1					
BUB1					
PMAIP1					
SMO					
CD226					
FLCN					
AKR1B10					
S100P					
ADORA3					
PTMA					
VTCN1					

Septin9					
MIR200B					
CCL22					
MIR125B1					
GREM1					
CASP10					
GAB2					
CARM1					
TNFRSF4					
ATG5					
NRP2					
MAP3K8					
BCL3					
HAX1					
SFPQ					
AXIN2					
CSNK1E					
MIR375					
NEK2					
STC1					
KIF2C					
TDGF1					
SEPP1					
TAS2R38					
AOC3					
RUVBL1					
PRKAR2A					
FOSL1					
PLCB1					

SLC22A2					
EIF5A					
PLAGL1					
PTGIS					
REG1A					
MIR150					
FOXE1					
KISS1R					
TAPBP					
MIR124-1					
MIR125A					
EXO1					
E2F4					
MIR200A					
FUT3					
SUV39H1					
ABCC3					
KRT20					
S100A10					
SAT1					
SIX1					
SUZ12					
MIR148A					
SMC1A					
ATP2B4					
CTNNBIP1					
NUDT1					
APOD					
TRIB3					

MIR10B					
MIR182					
HMMR					
RRM2					
PEX5					
PSME3					
PSMD10					
LAPTM4B					
NODAL					
LUM					
RPS6					
CLIC1					
ST6GAL1					
MBD2					
PTPRJ					
MTNR1A					
MARCKS					
PAK4					
NUMB					
UGT1A7					
AIMP1					
ACVR2A					
CLDN7					
MIR214					
FES					
MYBL2					
SLC46A1					
LIN28B					
LGALS3BP					

MIR206					
MAD1L1					
MIR141					
FOXO4					
MSI1					
NPR1					
ID4					
DPYSL2					
RAD18					
MAPRE1					
MPG					
POLE					
RALA					
MTAP					
TDG					
MIR15A					
PODXL					
PKHD1					
PLIN2					
HSPB2					
CBFB					
MSH3					
KCNH1					
MST1					
PELP1					
PLSCR1					
EGLN3					
GRP					
ETV4					

ITGB6					
F2RL3					
TRAP1					
SYP					
PPP1R13L					
MIR30A					
LATS2					
TPX2					
RACGAP1					
REG4					
SLC10A2					
B4GALT1					
LAMA5					
CXCR5					
TNFAIP6					
POLD1					
TAGLN					
TDP1					
IMPDH2					
APTX					
AMFR					
NCF4					
AKAP12					
RAD21					
CCR4					
GNA13					
GLS					
MEIS1					
CBX3					

MIR22					
RORC					
SFRP2					
CLDN3					
NNMT					
IL13RA2					
NDUFA13					
NEIL1					
ECT2					
T					
ESM1					
DVL1					
MIR218-1					
PTGDR					
MIR199A1					
ZBTB7A					
MIR451A					
CXCL14					
WWTR1					
PPP2R2B					
CEBPD					
FUT4					
XAF1					
TIA1					
KLK7					
MIR26A1					
AQP9					
MIR100					
ATP5B					

PSMD9					
LIFR					
RICTOR					
MMP11					
WISP1					
TARBP2					
LOXL2					
KDM5B					
PTGDR2					
MAL					
MT-ND2					
USP22					
MIR195					
HIF1AN					
LAMB1					
TJP2					
MIR181A1					
S100A11					
PIAS3					
WNT7A					
STK4					
CCDC88A					
SOCS2					
LRIG1					
PROK1					
SOX17					
SLC16A4					
IGFBP6					
CCAR2					

UBD					
TFF2					
DSC2					
GOLPH3					
FUT1					
AGA					
NR2F2					
MBNL1					
QKI					
MIR34C					
MYH11					
MIR132					
AIM2					
HYAL1					
PSMA7					
TET1					
ACTR2					
HTATIP2					
SLC1A5					
MMP26					
FGF8					
SYNE1					
TNFSF9					
CSE1L					
LASP1					
SRI					
FUCA1					
PRSS8					
ADAM8					

MEG3					
LCP1					
DDX6					
SLC22A3					
CHKA					
KLK10					
WARS					
MIR204					
MIR9-1					
CDC7					
DVL2					
ABI1					
PPP2R2A					
MIR19A					
HMGB2					
ACLY					
MIR1-1					
MIR224					
PLCD1					
FPGS					
MIR96					
WISP3					
SPINT1					
WNT2					
UMPS					
SEL1L					
MIR149					
CSMD1					
MIR93					

MIR34B					
BACH1					
SATB2					
HSPE1					
CRY1					
ADGRE5					
CRY2					
MIR23A					
EREG					
MIR29C					
ACY1					
GGH					
SLC7A11					
MIR335					
HNRNPH1					
RBFOX1					
TKTL1					
CPE					
GIPC1					
NLK					
RND3					
CDH17					
FRZB					
SELENBP1					
HVCN1					
GSTO2					
SPOP					
PINX1					
HIC1					

MIR29B1					
ELF3					
PCDH10					
CYTH2					
MAGEA1					
THBS4					
CTSH					
OLFM4					
COL6A3					
PIWIL1					
SLC26A2					
ABCC5					
LRAT					
DCLK1					
UGT1A					
RECQL5					
ATF1					
POLR2F					
TPD52					
ADH7					
PITX1					
EED					
EPHA1					
MIR133A1					
GUCY2C					
STC2					
IFITM1					
VASH1					
PLS3					

BUB3					
EPHB6					
TWIST2					
TIMP4					
PPP2R1B					
AURKC					
PHLPP1					
CASP4					
MIR24-1					
OSMR					
DSC3					
MIR378A					
DAB2IP					
STOML2					
DBP					
KLF8					
ANGPTL2					
SULF2					
ARID1B					
PTK7					
ARHGEF12					
SPHK2					
HNRNPF					
TACR2					
MIR494					
MELTF					
ANTXR1					
HNRNPM					
SEMA3F					

PLA2G10					
MIR25					
RYR3					
ZNF148					
CES2					
PBK					
GAS5					
RPLP0					
TCF7					
MAP2K5					
CNOT7					
CASP8AP2					
MIR18A					
EPHA4					
ZNF217					
GATA5					
WNT10A					
ABCB5					
LGMN					
TRG					
EPHB1					
NPAS2					
SULT2B1					
EGFL7					
DUT					
MIR196A1					
KLF9					
ATOH1					
MIR133B					

ST13					
ADM2					
MIR7-1					
DYX1C1					
DTL					
SGO1					
ATG7					
ANXA11					
MYCL					
MCRS1					
TUBB4B					
HYAL2					
NOB1					
MGLL					
MIR144					
MIR140					
MIR185					
SESN2					
NEAT1					
DPP6					
MLH3					
FOXF1					
KMT2C					
ADRM1					
ZFX					
RASSF2					
DVL3					
ERBIN					
DSC1					

COPS6					
KLK11					
SLC7A1					
DUSP4					
TFDP1					
HEY1					
SLC5A8					
ACO2					
TMPRSS4					
MIR92A1					
ERP29					
UVRAG					
SLCO2A1					
CLIC4					
NTHL1					
CDKN3					
FOXQ1					
WISP2					
MIR192					
IL13RA1					
MIR23B					
CDK2AP1					
NUP88					
MIR139					
CTNNA3					
MIR106A					
CIAPIN1					
SIRT7					
MIR302A					

PARVA					
PRKAG1					
MGEA5					
ADAM28					
PCGF2					
COL4A6					
WIPF1					
SERPINA10					
PROK2					
FZD7					
PARVB					
WASF3					
MIR212					
CDX1					
MIR128-1					
UCA1					
CASP5					
GSPT1					
CEACAM7					
ABHD5					
MIR320A					
CACYBP					
SNX9					
PIWIL2					
RGS6					
LIMK2					
MIR429					
C1GALT1C1					
PIK3R3					

VNN1					
MIR99A					
RALB					
HSPB6					
SPG20					
TXNDC5					
EYA4					
KDM3A					
EFEMP2					
GAS1					
VTI1A					
MIR138-1					
MIR497					
TRO					
GPX2					
PDHX					
C2orf40					
UBE2M					
BIRC6					
NRCAM					
MIRLET7C					
MMP16					
SETD1A					
XPO5					
KLK14					
BRAP					
SCG5					
RNF41					
BPTF					

PUF60					
MIR193A					
CDCA8					
MIR503					
MELK					
LRG1					
VANGL1					
SIPA1					
RGS16					
SLC16A7					
MIR130B					
NOL3					
PMS1					
FGF20					
ECHS1					
SEMA3B					
ORM2					
AKAP10					
SF3A1					
FZD3					
MED19					
HBP1					
GALR1					
RIN1					
IST1					
PAK5					
EMP1					
USP4					
SYNE2					

KIF18A					
RBM3					
MZF1					
PLA2G3					
EIF3H					
CTSZ					
CEACAM3					
NUCKS1					
CCL25					
PABPC4					
SUGT1					
MIR135B					
ATP5J					
CTHRC1					
POLD3					
UNC5C					
CYP2S1					
ZBTB7B					
HPGDS					
TNS4					
CLDN18					
TMEM8B					
ACSL5					
LGALS4					
GRHL2					
LMO1					
MIR338					
LIMA1					
SMUG1					

MGST1					
MT1F					
RNF43					
SCGB2A1					
MIR196B					
E2F5					
RPS6KA6					
MIR199A2					
S100A14					
KIN					
SPARCL1					
RAD54B					
RNF111					
NEIL2					
CDK5RAP3					
RETNLB					
LLGL1					
THOC1					
DUSP10					
KDM4B					
CEMIP					
SOX7					
MEP1A					
LZTS1					
LHX4					
RRBP1					
FUT6					
MIR215					
ABCF1					

ND2					
MT1G					
CBX7					
CYP2W1					
ATAD2					
ARL11					
SEC61B					
RGMA					
HOXB6					
MIR483					
GSTT2					
TFAP4					
ST8SIA2					
ZMIZ1					
PTPN14					
GLRX3					
RMI1					
BMP3					
MCC					
BAMBI					
CST1					
MIR124-3					
KIF2A					
NPRL2					
TPBG					
GCNT2					
AMBRA1					
ASCL2					
SUCLA2					

PTP4A2					
MTUS1					
SOCS6					
PTGES2					
MIR148B					
PTBP2					
GUCA2A					
SIRT4					
BCAT1					
FAM83H					
PRRX1					
HSP90AA2P					
GJC1					
MYO1A					
MMP21					
BLCAP					
NKX2-3					
PTPRR					
MIEN1					
DUSP22					
SRSF10					
FAN1					
VMP1					
CITED1					
MIR423					
LRRFIP1					
SYNJ2					
UHRF2					
BATF2					

PEBP4					
DKK4					
OTX1					
MOB1A					
EVL					
PDLIM1					
STYK1					
NDRG4					
LYPD3					
ARNTL2					
ADAMTS12					
ZG16B					
AJUBA					
ATG4B					
MIR135A1					
MIR211					
MIR129-2					
EPB41L2					
MIR218-2					
PES1					
SRPX2					
ADAM23					
HYAL3					
SSPN					
IL36A					
TUBB6					
ARID2					
FMNL2					
RAB22A					

MIR24-2					
AKAP4					
DIS3					
PHLPP2					
TMEM97					
MIR197					
RCC2					
UPK1A					
MIR301A					
SLC39A14					
GDF11					
EFNA2					
MIR340					
RPL18					
ADCY2					
EEF1E1					
WHRN					
MIRLET7I					
LMTK3					
CPEB4					
TNIK					
PSAT1					
MIR124-2					
PLA1A					
MED13L					
SH3GL3					
MIR372					
SEMA4A					
PRLHR					

RFXANK					
PATZ1					
MED28					
GINS3					
CD3EAP					
TNS3					
TOM1L1					
ANGPTL6					
FAM3C					
BTF3					
MIR9-2					
PLAGL2					
CST5					
PFDN1					
NMNAT2					
RAD51AP1					
MIR9-3					
NEUROG1					
TRAK1					
SGPP1					
MANBA					
MIR365A					
IP6K2					
SIK3					
MIR675					
TBPL1					
MIR153-1					
FAM134B					
GMDS					

MIR219A1					
TPST1					
CCAT2					
PPP1R3C					
TNFRSF19					
CDH10					
MIR1-2					
HIPK1					
BANCR					
REPIN1					
PDGFRL					
TAF1C					
GCNT3					
FGF6					
CLDN8					
MIR367					
SAPCD2					
TESC					
SLCO3A1					
MIR1246					
ZNF703					
FAM46A					
SLC35C1					
CBX8					
MIR129-1					
ATG10					
MIR491					
SCUBE2					
SYTL1					

PGPEP1					
WNT6					
HAO1					
HSPA4L					
LYAR					
B3GNT8					
DNPEP					
PFDN4					
FAM3B					
APMAP					
MIR608					
ASB9					
NEK11					
NAF1					
MIR32					
CHSY1					
MYNN					
CDK20					
MPP3					
ADAMTS14					
CLDN12					
RHPN2					
TENM3					
MIR20B					
MIR103A1					
MIR28					
PRIMA1					
B4GALT3					
ADAMTSL3					

DHX32					
KRT23					
SLFN11					
MIR154					
NME3					
SCRN1					
STK17B					
EPDR1					
MIR29B2					
SAT2					
TM4SF4					
BICC1					
MYRF					
DPPA2					
MAGED4					
GINS1					
PHACTR3					
ZKSCAN3					
HOXB8					
DESI2					
NXN					
HECA					
RPS27L					
SETD1B					
NR2F6					
RHBDD2					
MIR650					
PCDHGC3					
CSMD3					

CREB5					
MIR133A2					
CITED4					
GALNT12					
DDX46					
NOC3L					
MIR630					
LCORL					
HSPA12A					
PLEKHG6					
NKD1					
SMAP1					
ARHGAP6					
SLCO4A1					
CA11					
UBXN11					
TTYH2					
CBLN4					
CHPF					
MIR330					
CLDN23					
CDH22					
MIR92A2					
MIR99B					
THSD1					
CMTM2					
CACUL1					
ATP11A					
BTG4					

AFAP1L1					
DIP2B					
CRNDE					
SPIDR					
SHROOM2					
B9D2					
PCBD2					
CNPY2					
TUSC7					
MIR874					
DSCC1					
SLC25A28					
ACOXL					
TNN					
CHPF2					
LPCAT4					
TMEM25					
MIR584					
TFAP2E					
ZNF292					
CATSPER3					
FSTL5					
MIR26A2					
MIR605					
PARP11					
WDR76					
MORC1					
WSB2					
MIR498					

MIR103A2					
CCSER1					
CASC8					
SUSD6					
C2orf68					
MIR345					
CHSY3					
DNAJB1P1					
MIR454					
MIR484					
KIAA1456					
FAM173B					
MIR612					
ARHGAP30					
MIR374B					
LACTB2					
LONRF1					
MIR320B1					
GPATCH1					
MIR520G					
DACT3					
PNMAL1					
HCFC1R1					
MIR760					
MFAP3L					
CABLES2					
MIR422A					
ZNF768					
MIR1297					

PARP6					
C5orf66					
RERGL					
SEC1P					
CT47A11					
MIR551A					
MIR520A					
MIR1307					
FAM83E					
ZNF492					
TMEM200A					
LINC01475					
AMER3					
MIR552					
MIR592					
MIR601					
CRCS2					
ZNF700					
MIR1288					
CRCS9					
CRCS11					
CRCS5					
CRCS6					
MIR103B2					
MIR103B1					
C8orf37-AS1					
SRSF10P2					
NCRUPAR					
CRCS10					

CRCS8					
CRCS7					
MIR518A2					
MIR518A1					
DNMT3AP1					
MIR587					
LINC01296					
NPM1P5					
ZMIZ1-AS1					
FLJ22763					
MRPL42P4					
RPL27AP8					
RPL21P46					
VN1R86P					
LOC100131315					
LOC101928844					
